# ‘Once I knew there was a choice, I wanted to exercise that choice': using qualitative methods to understand why patients decline surgical trials

**DOI:** 10.1186/1745-6215-14-S1-P9

**Published:** 2013-11-29

**Authors:** Emily Harrop, John Kelly, David Neal, Prokar Dasgupta, Gillian Basnett, Robert Huddart, Allan Barham, Colin Thompson, Angela Casbard, Hala Jundi, Gareth Griffiths, Annmarie Nelson

**Affiliations:** 1 Marie Curie Palliative Care Research Centre, Wales Cancer Trials Unit, School of Medicine Cardiff University, Cardiff, UK; 2 Wales Cancer Trials Unit, Scool of Medicine, Cardiff University, Cardiff, UK; 3 Department of General Surgery, University College London, London, UK; 4 Department of Oncology, University of Cambridge, Cambridge, UK; 5 Guys and St Thomas Hospital, London, UK; 6 The Royal Marsden NHS Foundation Trust, London, UK; 7 University College London Hospitals, London, UK

## 

The **BOLERO** trial (Bladder cancer: Open versus Lapararoscopic or RObotic cystectomy) is a pilot study to determine the feasibility of randomisation to open versus laparoscopic access cystectomy in patients with bladder cancer. We describe the results of an embedded qualitative sub-study which explored why patients decline randomisation.

## Methods

10 semi structured interviews were undertaken with patients recruited from 3 sites in England. Data were analysed for key themes.

## Results

Most patients declined the trial because they had preferences for, and could choose, which surgical method they would be given- in most cases the robotic option. Patients described an intuitive ‘sense' that favoured the new technology and had carried out their own inquiries, including internet research and talking with previous patients and with friends and family with medical backgrounds. Medical histories and lifestyle considerations also shaped these personalised choices. Of importance too, however, were the messages patients perceived from their clinical encounters. Whilst some patients felt their surgeon favoured the robotic option, others interpreted ‘indirect' cues such as the ‘established' reputation of the surgeon and surgical method and comments made during pre-op assessments. Many patients expressed a wish for greater direction from their surgeon when making these decisions.

## Conclusion

Patients like to exercise informed choice over the type of surgery they receive. For trials where the ‘new technology' is routinely available to patients, there will likely be difficulties with recruitment. This questions the feasibility of similar trials in the future.

